# Alignment of gene expression profiles from test samples against a reference database: New method for context-specific interpretation of microarray data

**DOI:** 10.1186/1756-0381-4-5

**Published:** 2011-03-31

**Authors:** Sami K Kilpinen, Kalle A Ojala, Olli P Kallioniemi

**Affiliations:** 1 Institute for Molecular Medicine Finland (FIMM), University of Helsinki, Tukholmankatu 8, Helsinki, Finland

## Abstract

**Background:**

Gene expression microarray data have been organized and made available as public databases, but the utilization of such highly heterogeneous reference datasets in the interpretation of data from individual test samples is not as developed as e.g. in the field of nucleotide sequence comparisons. We have created a rapid and powerful approach for the alignment of microarray gene expression profiles (AGEP) from test samples with those contained in a large annotated public reference database and demonstrate here how this can facilitate interpretation of microarray data from individual samples.

**Methods:**

AGEP is based on the calculation of kernel density distributions for the levels of expression of each gene in each reference tissue type and provides a quantitation of the similarity between the test sample and the reference tissue types as well as the identity of the typical and atypical genes in each comparison. As a reference database, we used 1654 samples from 44 normal tissues (extracted from the Genesapiens database).

**Results:**

Using leave-one-out validation, AGEP correctly defined the tissue of origin for 1521 (93.6%) of all the 1654 samples in the original database. Independent validation of 195 external normal tissue samples resulted in 87% accuracy for the exact tissue type and 97% accuracy with related tissue types. AGEP analysis of 10 Duchenne muscular dystrophy (DMD) samples provided quantitative description of the key pathogenetic events, such as the extent of inflammation, in individual samples and pinpointed tissue-specific genes whose expression changed (*SAMD4A*) in DMD. AGEP analysis of microarray data from adipocytic differentiation of mesenchymal stem cells and from normal myeloid cell types and leukemias provided quantitative characterization of the transcriptomic changes during normal and abnormal cell differentiation.

**Conclusions:**

The AGEP method is a widely applicable method for the rapid comprehensive interpretation of microarray data, as proven here by the definition of tissue- and disease-specific changes in gene expression as well as during cellular differentiation. The capability to quantitatively compare data from individual samples against a large-scale annotated reference database represents a widely applicable paradigm for the analysis of all types of high-throughput data. AGEP enables systematic and quantitative comparison of gene expression data from test samples against a comprehensive collection of different cell/tissue types previously studied by the entire research community.

## Background

Gene expression microarray data published by the entire biomedical community have been organized and made available for data mining in several public databases (e.g. Oncomine, Gene Expression Omnibus, Array-express, GeneSapiens) [1-7]. This has facilitated analyses of gene networks and gene regulatory processes [8-12], and the identification of tissue- or disease-specific gene expression patterns [13-19]. Comprehensive microarray databases could also provide a powerful reference for guiding interpretation of new microarray data produced from test samples [20]. Such an approach would be particularly appealing for the analysis and interpretation of data from individual samples. Here, we have developed a microarray data analysis approach based on the similar concept as the simple, yet highly powerful and versatile sequence alignment comparisons (e.g. BLAST) for matching an unknown test DNA sequence against a comprehensive reference database of previously sequenced samples. The Alignment of Gene Expression Profiles (AGEP) method compares expression profiles of individual test samples with reference data obtained from large public gene expression microarray databases that are normalized to allow direct quantitative comparisons with the data from the test sample. The method provides the likelihood of the profile representing each of the known reference profiles as well as the sets of genes that show concordant and discordant expression levels against each of the reference datasets. Here, we describe the AGEP method and validate its utility in the analysis of microarray data from normal and disease tissue types as well as the quantitative analysis of cell differentiation patterns.

## Results

### Description of the AGEP method

We have created a tool to facilitate the comprehensive analysis and interpretation of gene expression profiles from individual test samples by comparing them against a reference dataset of previously analyzed, well-characterized and annotated samples from different tissues, pathologies, cell types or treatments. The AGEP method is based on the use of kernel density estimates for the expression levels of genes across each of the reference sample types (e.g. tissues). Density estimates make it possible to determine which gene expression states are characteristic for each gene in each tissue type, and can be used to compare individual test samples against the reference data.

To illustrate the AGEP approach, we used a reference dataset consisting of normalized Affymetrix gene expression microarray profiles from 1654 normal samples corresponding to 44 distinct healthy tissues types from the GeneSapiens database [7]. The 1624 samples contained data for 6290-17220 genes, depending on the Affymetrix array generation used. All available genes were used in the analysis. On average, each tissue type was represented by 37 samples (Additional file 1). Obviously, any similar unified dataset could be used as reference data for the AGEP method. The GeneSapiens data arise from several different Affymetrix array generations that were normalized to universal expression units to generate a single unified dataset comparable across the sample types. For further description of the data or the normalization, see [7,21]. All the individual test samples were similarly normalized to make them comparable against the reference data.

For each gene in each tissue type in the reference data, we first calculated the density estimate of expression values between zero and the maximum observed value in the entire reference data,(Additional file 2A-B) using kernel density estimation. This resulted in both gene- and tissue type-specific density estimates. Approximately 16% of the genes had a bi- or multimodal distribution in the reference tissues highlighting the importance of using density distributions as a base for the AGEP analysis.

**Figure 1:**
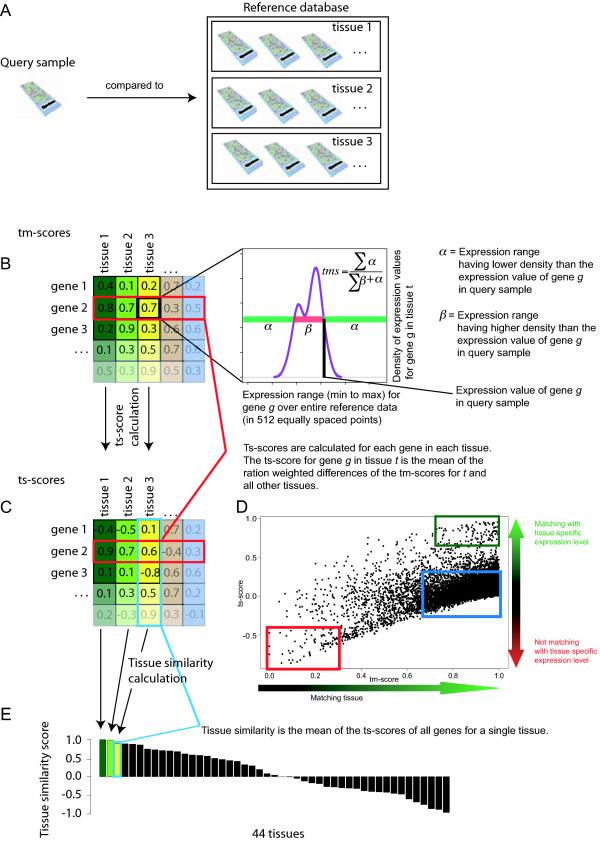
**Principle of the AGEP method, comparing microarray data from one test sample against a large reference database of different tissue/cell types**. A) The expression profile of a test sample is first normalized to be compatible with reference data. Density estimates are then calculated for expression levels of each gene in each reference tissue type. B) Data for each gene in the test sample is aligned with the density estimates of all the normal reference tissue types to calculate an tissue match score (tm-score). This defines the likelihood that the expression of the gene originates from the reference values, with the score of 1 indicating that the gene in the input sample had the best match with the levels for that tissue type. A tm-score of 0 means that the input sample had an expression level that did not match the reference tissue type at all. C) Tissue specificity scores (ts-scores) for each gene of the test sample for each tissue in the reference database are then calculated from the tm-score matrix (see methods). Ts-scores range from -1 to 1 and indicate how uniquely the test sample resembles a certain tissue type according to the gene's expression level. D) Scatter plot visualization of tm- and ts-scores of all genes for a single query sample against one reference tissue type. Genes highlighted with the green box have matching and tissue specific expression level in the reference tissue and the query sample, genes highlighted with a blue box do not have tissue specific expression level in this tissue but the expression level of the query sample matched that, genes highlighted with a red box have tissue specific expression level in this tissue but the expression level of the query sample did not match that E) Based on the mean of ts-scores for all genes for each reference tissue type, similarity of the test sample against all the reference tissue types is displayed as a bar graph.

After transforming the entire reference dataset into density estimates, data from individual test samples can be compared against the density estimates of the reference data (Figure 1A). In order to achieve this, we first quantify for each gene how well its expression level in the test sample matches the levels seen in each of the tissue types in the reference data. This similarity is defined as the tissue match score (tm-score) for each gene in each reference data tissue type, ranging from 0 (no match) to 1 (perfect match). The tm-score is defined by calculating the proportion of the expression range for a gene where the density estimate in a particular reference tissue type is lower than the value of that gene in test sample (Figure 1B). It can be thought of as the likelihood that the test sample's value matches with the most frequently observed expression range for this gene in that specific tissue type. For example, if a gene is expressed in the test sample at a level which has the highest density value in a reference tissue type, then the tm-score for that gene is 1 for this reference tissue type. Therefore, based only on this one gene, the test sample matches the reference tissue perfectly.

Tm-scores (Figure 1B) define how well the expression values in a test sample match with each of the reference tissue types however they do not define how unique, or tissue specific, those matches are among the various reference tissue types. In other words, a gene in a test sample may have an expression value with a perfect match (tm-score of 1) against a reference tissue, but compared to the tm-scores of other reference tissues, this match may be completely unique or not unique at all (Additional file 2C).

To find out this uniqueness, we calculate tissue specificity scores (ts-scores) (Figure 1C). These are formed by comparing the tm-scores (Figure 1B) of a gene among all the reference tissues types. For this purpose, we take the mean of the ratio of the weighted differences between the tm-score of a single tissue and the tm-scores of tissues. For example, in the Figure 1B, the tm-scores for gene 2 (highlighted in red) are compared to find out how much the tm-scores for each reference tissue type differ from the tm-scores of other tissue types. This results in ts-scores for gene 2 for all reference tissue types as highlighted in red in the Figure 1C. Ts-scores vary between -1 and 1. A ts-score of 1 for a gene in a reference data tissue means that the test sample had an expression level of the gene that perfectly matched the reference tissue (tm-score 1) but did not match at all any other reference tissue (tm-scores close to zero for all other reference data tissues). This means that the test sample had an expression level for the gene which is very specific for the tissue type and therefore provides a strong indication that the test sample originates from that tissue (Additional file 2C). A ts-score of -1 means the opposite; i.e. the test sample did not match the tissue specific expression level of the reference data tissue in terms of the gene in question.

The comparison of individual test sample against the reference tissue types leads to a matrix of tm-scores (Figure 1B) and a matrix of ts-scores (Figure 1C). The interpretation of both these scores for one individual test sample is summarized in Figure 1D showing for all genes how good the match was (tm-scores on the x-axis) and how unique the match was (ts-scores on the y-axis). Genes highlighted in green have both high tm-scores and high ts-scores meaning that the test sample's expression levels for those genes both matched with that reference tissue type (high tm-score), and that this match was also unique to that tissue type (high ts score). Genes highlighted in red are such that they have a tissue specific expression level in the reference data tissue in question but the expression values in the test sample did not match those. Their tm-score for the reference tissue in question were very low, and the tm-scores for other tissues were high, thus the ts-score ended negative. Genes highlighted in blue have high tm-scores meaning that these genes' expression in the test sample matched well with the reference tissue type, but that these expression levels also matched with many other reference tissues, implying little or no uniqueness (ts-scores around zero). Both the tissue match (tm) and tissue specificity (ts) scores can be used to interpret the nature of a test sample. One such interpretation is to calculate the average of the ts-scores for each of the reference tissue types (Figure 1E). This tissue similarity score can be used as a metric to identify the tissue of origin of the test sample.

Detailed methods and formulae are provided in the methods section.

### Comparing AGEP with existing methods

The idea of using existing microarray data to identify or categorize a new external sample is not new. Many scientists are using unsupervised clustering methods, such as hierarchical and k-means, to understand relationships between samples. Unsupervised clustering is considered as a simple, yet effective method. However, if the reference data are complicated and do not cluster according to their annotation, classification of the outside sample is challenging if not impossible.

In comparison to existing methods, AGEP method can be termed a search & retrieval based method comparing single or multiple query samples against a reference database [22-24]. Search & retrieval methods not only try to identify most similar reference group, a task of traditional classifiers like nearest-neighbor (NN) [25,26] and support vector machines (SVM) [27-29], but also to provide interpretation of the component-wise (e.g. gene-by-gene) contributions to the similarity match.

AGEP performance in tissue identification task with both leave-one-out cross-validation (LOOCV) [30] of the entire reference database and with an external dataset was compared to both a nearest-neighbor classifier [25,26], traditional instance-based learner, and to SVM [27-29], more complex algorithm with good classifying performance. These both are supervised clustering methods, suitable for tissue identification tasks and therefore suitable for benchmarking AGEP performance in the same task.

In LOOCV of the entire reference database AGEP reached overall accuracy of 93.6% (with a range of 58.3-100% depending on tissue type) (Additional file 3, Table 1). Average sensitivity for the identification of tissue type of origin was 0.925 and average specificity 0.998 (Additional file 4). Secondary matches to other tissues often reflected known anatomical and biological similarities (Additional file 5).

**Table 1:** Accuracy of the AGEP method to find *a priori *known annotation class as primary hit in leave-one-out cross validation of the entire reference database against itself and accuracy of the SVM to find *a priori *known annotation class in 10-fold cross-validation of the entire reference database

	AGEP Accuracy	Nearest-neighbour (correlation)	SVM Accuracy
**Max**	100%	100%	100%

**75% percentile**	100%	100%	100%

**Median**	96.4%	93.7%	96.7%

**Mean**	93.7%	90.7%	90.4%

**25% percentile**	90.3%	81.5%	91.7%

**Min**	58.3%	69.2%	9.1%

**Overall**	93.6%	90.2%	94.4%

LOOCV of the entire reference database with nearest-neighbor (NN) classification produced 65.1% overall accuracy with Euclidean distance, and 90.2% with Pearson correlation coefficient (Table 2). SVM resulted in 94.4% overall accuracy in 10-fold CV (Table 1) of the entire reference database. 10-fold CV, another well established way to evaluate classifier performance [30], was chosen instead of LOOCV for SVM due to the computational requirements of SVM. Median imputation for missing values was used, which was necessary with SVM as virtually none of its implementations can handle missing values. This potentially enhanced the performance of SVM as the within tissue variation for median imputed genes was considerably lower than for non-imputed genes. Additionally, due to its constraints concerning missing data, SVM was run using only 11 834 genes of the 17 225 present in the data.

**Table 2:** Summary of the tissue identification capabilities of most related methods

Method	Strengths	Limitations	LOOCV (or 10-fold CV)	Independent validation
AGEP	Good classifier. Results available per gene, with a biologically meaningful distance metric.	Computationally intensive. Weight of all genes equal.	93.6% accuracy	96.9% combined accuracy

NN	Relatively robust and easy to setup.	Very sensitive to the selection of parameters and the distance metric chosen. No simple choice for distance metric. No simple way to interpret gene-by-gene contribution to the similairy.	90.2% accuracy	94.4% combined accuracy

SVM	Powerful classifying performance if properly customized for the task	No simple solution for selection of kernel. With complex tasks somewhat subject to overfitting. No gene-by-gene contribution available in biologically interpretable manner.	90.4% accuracy NOTE: due to computational limitations was actually 10-fold cross-validation.	98.0% combined accuracy

DNA barcode (Zilliox et al. 2007)	Good classifier. Simple to understand per gene comparison.	Per gene classification is binary, missing out a lot of the variation.	Not tested	Not tested

Cancer molecular classification (Parmigiani et al. 2002)	Good classifier. Simple to understand per gene comparison.	Per gene classification is ternary, missing out a lot of the variation.	Not tested	Not tested

Probabilistic retrieval and visualization of biologically relevant microarray experiments (Caldas et al. 2009)	Good at finding experiments that repeat biological responses.	Works for gene sets derived from comparative experiments	N/A	N/A

We then proceeded to compare the performance of all three methods with an external dataset of 195 healthy tissue samples from the Array Express [1] study E-GEOD-7307. Overall accuracy of the AGEP method to identify tissue of origin within this dataset was 96.9%, with 84.6% matching the exact tissue type and another 12.3% matching closely similar tissue types. In fact, all of these similar tissues were from the central nervous system and represented different anatomical parts of the brain. Therefore, only 3.1% of the external samples were identified incorrectly in terms of the tissue type (Additional file 6). With the same external dataset nearest-neighbour method (with Pearson correlation coefficient as distance measure) resulted in 78.3% accuracy to the exact tissue, and another 16.1% matching a similar tissue, leaving 5.6% of the samples incorrectly identified. SVM resulted in 98.0% overall accuracy.

The nearest-neighbour classifier achieves almost the same absolute accuracy than AGEP, but it has serious limitations. As highlighted by the LOOCV results, the choice of distance method greatly affects the results, while no biologically reasonable single distance method exists. Other commonly used instance-based learners, as k-nearest neighbor (k-NN), are also very sensitive to parameter selection. In contrast to AGEP, there is no simple way to understand the individual genes' contribution to the similarity. SVM offers a high accuracy as well, but does not offer gene-level data on the similarities either. Also, SVM methods are better suited to binary classification tasks, rather than choosing the correct group from a multitude of options. Ensembles of SVM classifiers have been successfully implemented for complex classification tasks, but they have a known tendency for over-fitting and usually require complex and difficult case-by-case selection of the optimal kernel [31].

A recently published method by Caldas et.al. [23] provided 82% accuracy for identification of biologically relevant experiments when queried with data from external experiments. This method uses gene set enrichment, not individual gene expression, as the basis of its similarity. Therefore, data from individual samples cannot be analyzed, and the categories are experiments where a comparison between two sample sets is needed. This method also collapses the gene expression values by medians, thereby not addressing the problem of multimodal gene expression distributions, which AGEP was specifically designed to solve.

Other classification methods that operate per gene do exist, such as molecular classification of cancer [24] and gene expression barcode [22]. These methods have been found to be accurate in determination of tissue type, but they bin the genes' expression profiles into on/off (bar code) or downregulated/normal/upregulated (molecular classification) before using them for classification purposes. AGEP also operates on a per gene basis, but the way of looking at the expression profiles in the sample categories differs fundamentally from the abovementioned methods.

Overall, these comparisons indicate (Table 2) that AGEP performs the tissue identification at least as well as the existing classification and search & retrieval methods, while having the advantages that AGEP can i) compare a single query sample against a reference database ii) take into account bi- and multimodal expression profile in reference sample sets iii) deal with bi- and multimodal expression profiles, thereby more accurately reflecting the actual gene expression variability of *in vivo *samples iv) provide biologically important gene-by-gene interpretation of the similarity against multiple references v) handle missing datapoints.

### Biological interpretation of the gene-by-gene contribution to the similarity match

As AGEP data for each gene is biologically interpretable we then evaluated and validated the method in the interpretation of actual biological experiments.

#### Interpretation of microarray data I: Dystrophic muscle

We analyzed data from ten Duchenne muscular dystrophy (DMD) samples against the 44 tissue types in the reference database. In all cases striated muscle was identified as the primary alignment (Additional file 7). Heart and tongue also showed significant similarities, with uterus and prostate both scoring positively, probably linked to the relatively high smooth muscle content. Interestingly, adipose tissue was also among the top four alignments for all samples. This may reflect the common mesenchymal origin of these tissues as well as the fact that dystrophic muscle tissues may contain larger than normal amounts of adipose tissue [32]. For patient number four, adipose tissue was the second best normal tissue match. This sample may have contained more adipose tissue than others due to the disease progression [32] or specific subtype of the disease [33]. AGEP identified both the genes defining the similarity to the striated muscle as well as those with adipose tissue. This reflects the power of AGEP to provide context-specific interpretation of microarray data.

**Figure 2:**
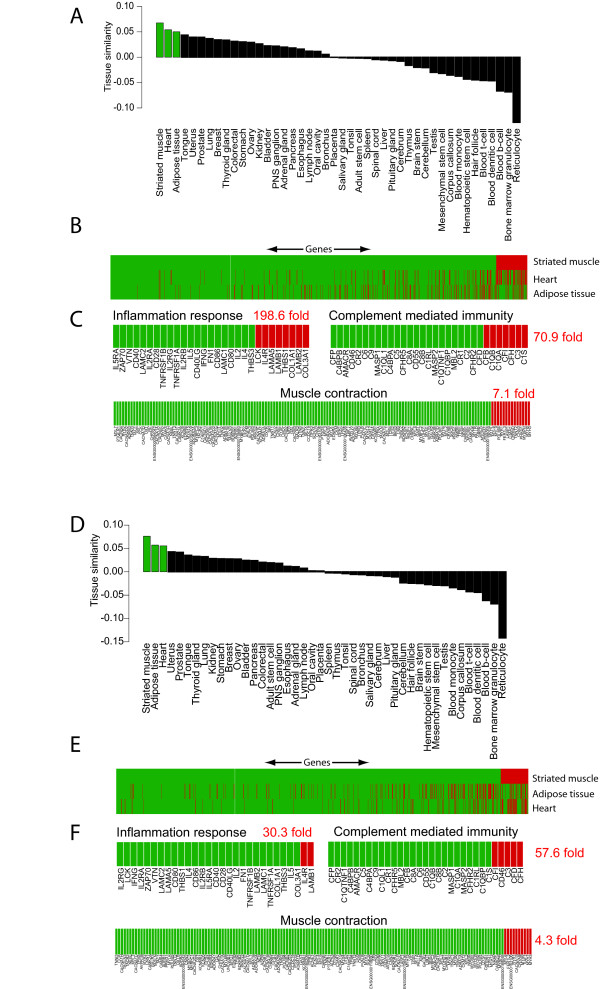
**Results of the AGEP analysis of microarray data from two Duchenne muscular dystrophy samples against the reference database**. A) The sample from patient 3 resembles most closely striated muscle among the 44 reference tissues. B) Alignment of the patient's transcriptome at the level of individual genes. On the x-axis are genes (17 330) and on the y-axis the three most similar tissues. Green color indicates that the genes have an expression level typical for that tissue, whereas red indicates atypical expression levels. Genes have been ordered according to their level of similarity against the most similar tissue (striated muscle). C) View of distinct gene sets and pathways for the most similar tissue (striated muscle). Relative enrichment of atypical genes is shown on the right side to illustrate aberrant gene expression levels for individual patient samples. Genes involved in inflammation response, complement mediated immunity and muscle contraction had more atypical expression levels as compared to healthy striated muscle (198.6, 70.9 and 7.1 fold enrichment of atypical genes, respectively), indicating that these processes were altered in DMD in comparison to healthy muscle. D-F) The gene expression profile from patient 4 resembled mostly striated muscle (primary match), but revealed adipose tissue as the second best matching tissue. As compared to patient 3, this patient had a larger number of muscle typical genes involved in inflammation response, complement mediated immunity and muscle contraction suggesting a less severe disease for patient 4.

AGEP analysis of dystrophic samples against healthy striated muscle reveals the disease-associated changes as as a decreasing level of alignment. For the sample from patient 3, gene sets with aberrant expression (Figure 2B-C) as compared to the reference striated muscle included inflammation, complement mediated immunity and muscle contraction (with 198.6, 70.9 and 7.1 fold enrichment of atypically expressed genes as compared to normal muscle, with a p-value < 0.05 for each). These are expected differences in DMD [32,34,35] and were seen for all other disease samples, with the exception of patient 4, (Figure 2D).

**Figure 3:**
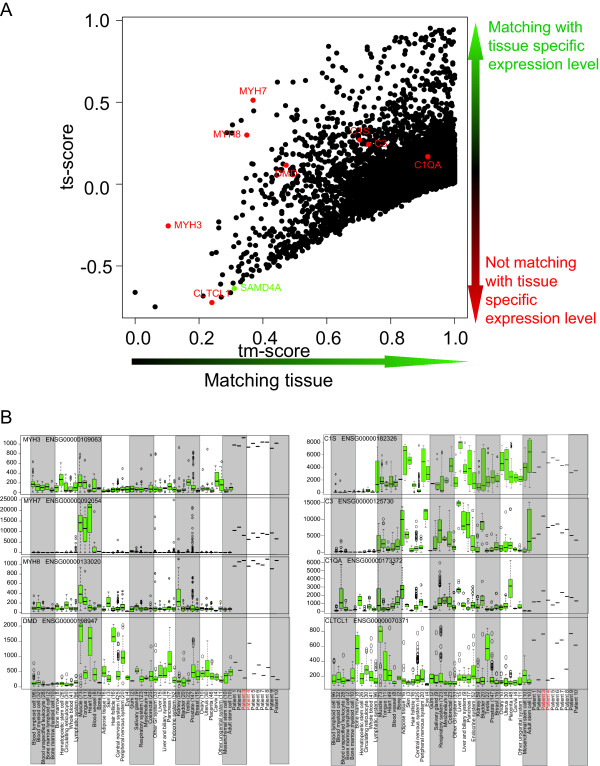
**A) Scatterplot of tm- and ts-scores of DMD patient (number 4) when compared against healthy striated muscle**. On the x-axis are tm-scores of the patient and on the y-axis are ts-scores of the patient. As explained in the figure 1D, upper right corner (high tm- and ts-scores) indicate genes having muscle specific expression and the query sample had expression level matching that. In this case there is clearly a group of genes having expression level assumed and specific for muscle and for this part the patients transcriptomic profile resembles healthy striated muscle. On the lower left corner are the genes (low tm- and ts scores) having muscle specific expression level (ts-score deviating from zero) but the query sample did not have expression level matching it. Thus these genes are potentially related to DMD. Eight *a priori *known DMD related genes are highlighted in red while novel gene in DMD (SAMD4A) is highlighted in green. SAMD4A has extremely muscle specific expression level but the query sample did not have expression matching it. B) Visualization of the normalized expression levels of selected genes from ten Duchenne Muscular Dystrophy samples in relation to the expression levels of these genes across all 44 normal tissue types. The green boxplot data display statistical data on gene expression for each of the normal tissue types (according to http://www.genesapiens.org), with the data from the 10 individual DMD samples added to the far right. Patients illustrated in figure 3 are colored red. *MYH3*, *MYH7 *and *MYH8*. *MYH3 *and *MYH8 *(left panel) are muscle contraction genes, whose expression is discordant for DMD tissue in patients 3 and 4. DMD samples show dramatic differences both between the patients and healthy striated muscle for the levels of expression of these genes. On the right panel are boxplots of genes *C1S*, *C3*, *C1QA*, representing key components of the complement mediated immunity process, whose expression values are different both between the patients and the healthy striated muscle. *CLTCL1 *is an interesting gene, whose expression is lost in 6 out of 10 patients.

We also explored the AGEP results at the individual gene level (Figure 3). First we selected five genes (*MYH7*, *C1S*, *C3*, *C1QA*, *CLTCL1 *and *DMD*) previously known to associate with DMD [33,32,36,37,35] and explored their alignment scores in individual patient samples. The dystrophin gene, *DMD*, a gene whose mutations underlie most muscular dystrophies [33], was underexpressed as compared to healthy muscle in all but one patient (patient 4) and scored a mean 0.37 as the tm-score. In contrast, *MYH3 *and *MYH8 *displayed overexpression in all patients, both being known hallmarks of dystrophic muscle [32,36], and received mean tm-scores 0.05 and 0.3, respectively. *MYH7 *had lower expression than seen in healthy striated muscle with a mean tm-score 0.5. *CLTCL1 *expression was heterogeneous, with four Duchenne patients having reduced expression levels that did not match muscle-typical levels with a tm-score of 0.28. In contrast, the mean of the tm-scores for the remaining patients was 0.79. *CLTCL1 *is involved in glucose transport in muscle tissue [38], a process known to be affected in the Duchenne dystrophy [37]. *C1S*, *C3 *and *C1QA *genes, involved in complement mediated immunity contributing to muscular dystrophy [35], also showed heterogeneous expression across the dystrophy samples, with corresponding changes in tm-scores. Having demonstrated the capability of AGEP to provide patient-specific alignment scores for the individual genes in a context-specific way, matching the previous biological knowledge on the disease biology (Figure 3), we then tested AGEPs ability to pick novel genes that have a muscle-specific expression which gets lost in the DMD disease samples. *SAMD4A *is highly muscle-specific gene, coding for a posttranscriptional regulator, but was among the 10 genes with the lowest ts-score of all genes in the DMD samples (the smaller the ts-score is the less gene matches the expression level unique for the tissue). S*AMD4A *had lost its muscle specific expression level in all dystrophy patients (mean ts-score of all patients -0.57). To our knowledge, loss of muscle specific expression of the *SAMD4A *gene has never been associated with DMD before.

As compared to other patients, patient number 4 had a unique disease with similarities to adipose tissue, less inflammation and immunity response, less impact on muscle contraction genes and dramatically reduced CLCTL1 expression (tm- ts-score scatterplot displayed in Figure 3A), giving a powerful example of the ability for AGEP analysis to rapidly reveal patient-specific characterization of molecular properties. The scatterplot identifies genes with a muscle specific expression pattern, and whether the query sample matched that expression or not. Genes with a low tm-score (doesn't match muscle) and a negative ts-score (matches other tissues better) reside in the lower left corner of the plot, indicating genes with muscle specific expression patterns that do not match the query. Similarly, genes with a muscle specific expression matching the query are located in the upper right corner.

Taken together, this DMD example indicates, how AGEP allows interpretation of transcriptomic profiles of individual patients at a level of tissues, biological processes and individual genes and will facilitate the molecular interpretation of microarray profiles from individual disease samples.

#### Application of the array alignment for the microarray data analysis II: stem cell differentiation

We then explored the AGEP method in the analysis and interpretation of transcriptional changes from a study of differentiating mesenchymal stem cells to adipocytes with three replicate samples measured over 5 time points (0 h, 1 h, 3 h, 9 h and 7d). Each of the 15 samples was aligned against 44 tissue types in the reference database to uncover transcriptional changes.

**Figure 4:**
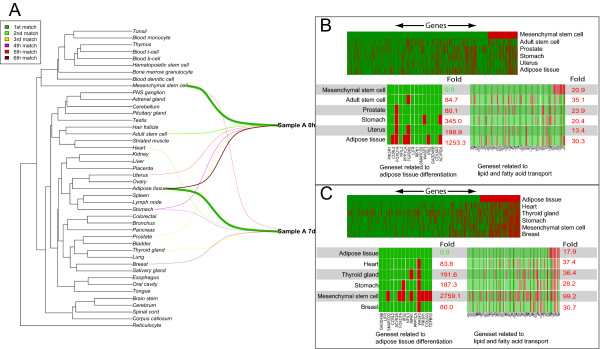
**Alignment of transcriptomes of two samples from a differentiation series of mesenchymal stem cells at the 0 h and 7d time-points**. A) Alignments for the two time-points are visualized in reference to all normal tissue types. On the left side is a phylogenic tree of all the 44 normal tissue types in the reference database arranged according to their similarities in gene expression against one another. Colored lines connect the test samples to their six best tissue matches, with the thickness of the line reflecting the number of typical genes. B) Heatmaps illustrating the AGEP results at the 0 h timepoint. The upper heatmap shows the alignment against all genes in the six best-matching tissues arranged in the order of their tm-scores with the best matching tissue. Green color indicates that the gene has a typical and red color an atypical expression level for each tissue type. The two lower heatmaps show results specifically for adipocyte differentiation and lipid and fatty acid transport gene sets. The relative enrichment of the gene set members among tissue atypical genes is shown on the right. C) Alignment results at the 7d timepoint. The data indicate that the transcriptomic program typical to mesenchymal stem cells decreased, while the cells gained adipose tissue like properties (panel B vs. C). At the 0 h time point all adipose tissue differentiation related genes are expressed at levels typical to mesenchymal stem cells with no resemblance to adipose tissue. At the 7d time point all these genes have acquired expression level typical to adipose tissues with no resemblance to MSC-style patter matching expression levels. Lipid and fatty acid transport gene set members show a similar tendency.

As anticipated, all the samples were initially similar to MSCs (Figure 4A, Additional file 8). Genes related to adipose tissue differentiation were expressed at the level expected for MSCs, and at an atypical level for adipose tissue (fold enrichment 1253.3 with p-value < 0.05) (Figure 4B). During the time series, AGEP analysis indicated how the transcriptomic program of the cells changed away from MSCs and gained similarity to adipose tissue. At 7 days, two samples already resembled adipose tissue more than MSCs. At this point, part of their transcriptome displayed heart-specific features as well. While the extent of this change was unexpected, *in vivo *derived MSC tend to differentiate *in vitro *to cardiac myocyte like cells [39].

Analysis of the biological processes involved (Figure 4B-C) indicates that all genes related to adipose tissue differentiation have acquired an expression level expected for adipose tissue, whereas a significant proportion (fold enrichment 2759.1, with p-value < 0.05) of these genes are no longer expressed at the typical MSC level. Similarly lipid and fatty acid transport genes have acquired expression values expected for adipose tissue, and a large number of them are now atypical for MSCs (110.6 fold relative enrichment with p-value < 0.05). In summary, during the differentiation, MSC-specific transcriptomic program is gradually lost and adipose tissue like program gained. However, the cells do not reach the full *in vivo *adipose tissue transcriptomic profile.

**Figure 5:**
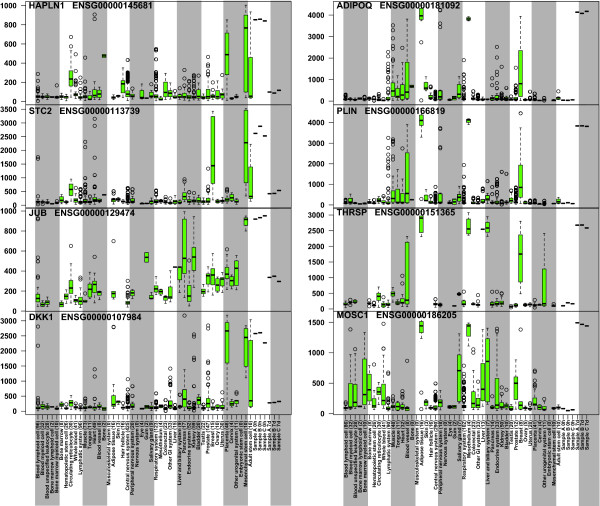
**Visualization of mesenchymal stem cell specific genes expression at time point 0 h (left column) and adipose tissue -specific genes at 7d time point (right column)**. Each boxplot shows the expression level of the corresponding gene in the reference data (from http://www.genesapiens.org) with the data from external samples (A, B and C) shown at the far right in the two time-points. *HAPLN1*, *STC2*, *JUB *and *DKK1 *have mesenchymal stem cell specific expression levels at the 0 h time point, while *ADIPOQ*, *PLIN*, *THRSP *and *MOSC1 *have high levels in adipose tissue. At the 7d time point the patterns is reversed.

We further studied the genes with the highest match to MSCs at the 0 h time point, and those with adipose tissue as the highest match at the 7d time point replicates (Figure 5). *HAPLN1*, *STC2*, *JUB *and *DKK1 *had the highest ts-scores for MSC similarity at the 0 h time point. *ADIPOQ*, *PLIN*, *THRSP *and *MOSC1 *genes all gained full adipocyte specific expression levels at 7 days, these genes are known to be adipose tissue related [40-43]. As a summary, AGEP analysis of the data on stem cell differentiation demonstrates the ability of the technology to quantitatively follow the gradual transcriptomic changes during mesenchymal differentiation, revealing both expected (stem cell to adipose tissue) and unexpected (heart tissue) differentiation, along with the identification of the specific gene expression differences in each comparison.

#### Application of the array alignment for the interpretation of transcriptome data from test samples III: hematopoietic cell types and myeloid leukemias

**Figure 6:**
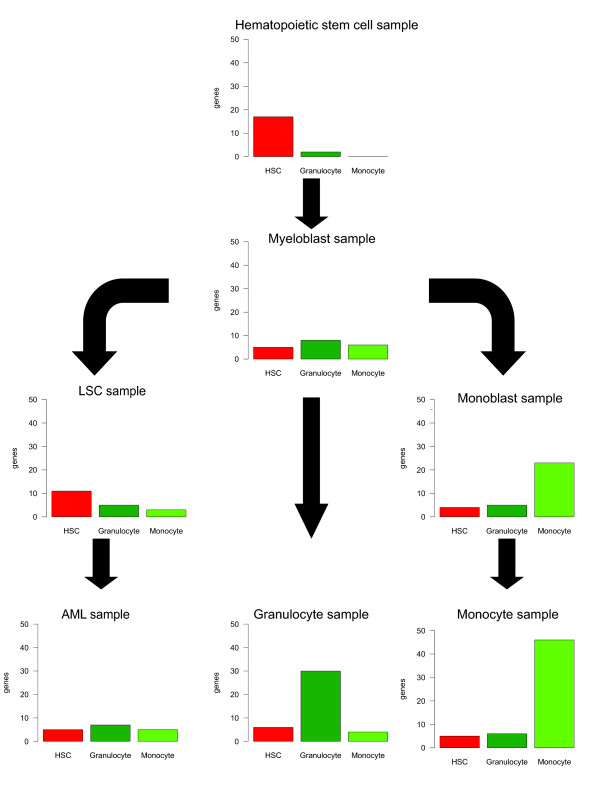
**Comparison of gene expression similarities based on AGEP analysis of seven samples representing various differentiation and/or malignancy states of myeloid cells (hematopoietic stem cells, leukemic stem cells, myeloblasts, monoblasts, AML, granulocytes and monocytes)**. For each sample, the number of tissue specific (ts-score >0.75) genes for the three reference tissue types (hematopoietic stem cell, bone marrow granulocyte, blood monocyte) is shown as bar charts. There is a gradual change in the transcriptomic program when moving from hematopoietic stem cells to the most differentiated granulocytes and monocytes as well as to malignant AML cells. As expected, the hematopoietic stem cell sample had several genes specific to the HSC reference cell type, while in the myeloblast sample this number is much lower. The myeloblast sample did not express monocyte or granulocyte specific genes. Leukemic stem cells have some HSC specific expression, while the AML resembles slightly more the granulocyte reference than the HSC or monocyte reference.

Data from seven cell types of the myeloid lineage: hematopoietic stem cells (HSC), myeloblasts, leukemic stem cells (LSC), acute myeloid leukemia (AML), granulocytes, monoblasts and monocytes were compared against the 44 tissue types of the reference data types. Figure 6 indicates the number of genes expressed in the test samples in a cell-type specific manner (ts-score >0.75) when compared against three specific sample types in the reference database (hematopoietic stem cells, granulocytes and monocytes). As expected, data from the hematopoietic stem cells were aligned most closely with HSCs in the reference database. Myeloblasts had roughly the same small number of cell-type specific genes corresponding to each of the three reference cell types. Monoblasts most closely resembled monocytes, but lacked specific genes expressed in the monocytic samples. Leukemic stem cells resembled HSCs the most, but with less HSC specific genes than the sample from the HSCs. The AML sample was further from the HSCs than LSCs, with some equally small similarity with both granulocytes and monocytes. Taken together, these data highlight the transcriptomic programs ranging from hematopoietic stem cells to mature myeloid cells.

## Discussion

A large number of methods have been developed for the analysis of microarray gene expression data, reflecting the tremendous complexity of the problem of transforming information on the expression levels of 20,000 genes into meaningful biological insights. Many microarray data analysis approaches are based on case-control study designs like comparing treated and untreated cells or matched disease and control tissues. However, the control group may be hard to define and challenging to acquire. In some cases, like with differentiating stem cells, multiple control groups would be needed in order to achieve a comprehensive understanding of the differentiation pathways. The method presented in this paper, AGEP, allows highly informative comparison of a single microarray sample against an existing reference database of annotated, previously analyzed microarray data.

The philosophy of AGEP is analogous to the sequence alignment methods in the analysis and comparison of newly sequenced DNA. These methods are highly powerful because of the availability of fully sequenced genomes and 108 million sequence records as a reference in the Genbank. The key difference between sequence-based and gene expression based methods is that the latter provides quantitative information, not just qualitative sequence identities. Therefore, we had to take into account distributions of gene expression levels in each reference tissue that are often multi-modal in nature. In the AGEP method, this was accomplished by calculating kernel density estimates for each gene in each reference tissue type, thereby generating reference data for characteristic expression profiles of all genes in all the major normal tissue types.

We feel that a simple categorization of gene expression into two or three categories (like underexpression, average and overexpression) is insufficient to capture the true behavior of genes. The way AGEP works is that we assume that the whole spectrum of expression values for a gene in a tissue reflects the true variation *in vivo*. Therefore, when we compare the expression value from an external sample to a reference database, we determine quantitatively how well that value fits the distribution in each reference tissue, instead of simply asking whether the gene is up- or down regulated in a direct comparison with a reference tissues, as these types of analyses are usually done.

One of the key features of the AGEP method is the tm-score. We believe that it is the best way to compare a single expression value to a host of values from any reference sample group, such as a single tissue. Unlike a single summary value (like mean or median), it is able to account for any type of expression distribution, and takes into account the observed expression range of the gene in question. It can also accommodate missing values, which is not the case for many other methods. It is also relatively robust against annotation errors as mixing two tissue types together will create a bimodal expression profile for at least some of genes and AGEP can accept that as a feature of the (mixed) tissue class whereas methods based single summary statistic would generate values that are not correct for either tissue types of the mix.

AGEP performance in finding correct tissue of origin for a set of samples was benchmarked by using both nearest-neighbor and SVM, the latter being one of the most powerful classifying engines available [27-29]. As AGEP reached at least similar performance levels as SVM, we do not anticipate that comparison to other methods would change the conclusion that AGEP's absolute accuracy in tissue identification is comparable to other key methods and adequate for most purposes.

For tissue classification purposes, tm-scores need to be evaluated in terms how well they differentiate each tissue from all the reference sample types. Transforming tm-scores to tissue specificity scores provides the necessary evaluation. The ts-score may not necessarily be the optimal method for testing the classification of the query sample against one tissue type. That being said, the high classification accuracy achieved by AGEP demonstrates that the tm-score is a good basis for comparing similarity of a single gene expression value to a reference pool.

Importantly, AGEP not only provides a metric of the sample similarities, but also defines the genes informative in comparison to all the reference tissues. This is important in order to understand the biological basis of the transcriptomic similarities. That is, rather than just asking the question "What tissues does this gene expression profile resemble?", AGEP can also answer questions like "which genes contribute to the similarity to a certain tissue?" or "what biological processes are different in the test sample as compared to the various tissues?", as evidenced by the presented case studies.

Previous methods for similar comparisons are typically based on an upfront selection of subsets of genes (gene sets or signatures) that are derived from the test samples and reference sets. Examples of conceptually similar approaches include the connectivity map [44,45], molecular concept mapping [46], and the relevancy metric [23], which all provide the capability to link new experiments to existing ones. Selected gene sets are most informative and powerful for the purpose they were designed for and depend entirely on the identification and annotation of meaningful gene sets that may or may not be available for a particular study. Also, gene sets may not transfer well from one context to another, e.g. from one tissue to another. Other informative gene expression patterns may be missed when focusing on gene sets or molecular concepts. AGEP does not depend on *a priori *assumptions of subsets of genes being more informative than others and it was designed to be used for the analysis of individual samples.

The AGEP method is widely applicable, but is particularly powerful when a deep interpretation of microarray results is needed for samples for which an optimal control tissue is not available due to technical, medical or biological considerations, such as cell differentiation and stem cell research, where comparisons with multiple different cell and tissue types are needed.

When selecting the reference data, we omitted any tissue with less than six samples. Obviously, human normal tissue specimens are hard to obtain in large quantities. Therefore, five is less than optimal as a statistical lower limit, as individual samples have a huge impact on the shape of the kernel density with so few samples. As more data become available, we would suggest raising the low limit to at least 20 samples, so that each reference sample type would have the representation of the spectrum of likely expression levels.

The computational requirements for AGEP are rather heavy, as the representation of the expression distributions as density estimates requires considerable amounts of memory. With the current implementation AGEP needs be run in a server with more than 10 GB of memory, however this is largely dependent on the size of the reference database used.

## Conclusions

Alignment of samples from Duchenne muscular dystrophy (DMD) patients revealed known critical and causative expression changes in the transcriptome of dystrophic muscle. For example, the well-known role of inflammation in dystrophy was clearly flagged by the AGEP analysis [33]. Known dystrophy related genes like *MYH3*, *MYH7*, *MYH8 *and *DMD *[32,33,36] and genes previously unlinked to the dystrophic muscle, such as the SAMD4 were identified by AGEP as having expression levels in dystrophic muscle not matching healthy muscle. Interestingly, *CLTCL1*, a gene related to glucose metabolism, was expressed at levels matching those in normal muscle tissue in 6 dystrophy patients while 4 had clearly lower expression illustrating how AGEP can provide interpretation of molecular profiles of individual patients, and reveal pathogenetic genes and pathways in a context-specific manner. Furthermore, as more annotated reference data becomes available, this will facilitate molecular stratification of patients suggesting many possible future applications in diagnostic molecular pathology.

In the examples on cell differentiation, the AGEP method facilitated understanding of the changes in the transcriptomic programs of stem cell differentiation to adipose tissue. Most MSC-specific genes (e.g. *HPLN1*, *STC2*, *JUB *and *DKK1*) lost their specific expression levels and acquired levels typical for adipocyte while adipocyte-specific genes (e.g. *ADIPOQ*, *PLIN*, *THRSP *and *MOSC1*) gained expression typical for adipocytes during the differentiation. Illustrating the key advantage of AGEP method in context-specific comparisons, we were able to identify that during the stem cell differentiation cells also gained similarity with cardiomyocytes. This differentiation pattern is well known [39], but the extent to which this takes place during adipocytic differentiation has not been comprehensively characterized before. AGEP also helped to unravel genes with unique expression levels in cell types of the myeloid differentiation cascade. These analyses quantified the cellular differentiation states (and genes involved) that could in the future be applied for developing diagnostic applications in mapping differentiation states of normal and pathological hematopoietic lineages or any other cellular differentiation cascade. In conclusion, our biological validation experiments showed that AGEP is capable of identifying gene-by-gene contributions to the similarity between query sample and reference database.

Even though tissue classification was not the primary aim of the study, the AGEP method achieved high accuracy in identifying the tissue type of origin of test samples and the biological processes and genes behind such similarities, thus facilitating understanding of biological concepts hidden in the complex transcriptomic profiles. Future implementation of this line of research could lead to diagnostic approaches for analysis of unknown primary tumors.

Taken together, the AGEP methodology provides a new paradigm for comprehensive analysis of gene expression profiles from individual samples, making efficient use of existing knowledge and collective data acquired by the research community. This AGEP concept is similar to the widely applied sequence alignment tools, where a new test sequence is compared against a large reference collection of known genomes and sequence repositories. We therefore believe that the AGEP approach will incrementally gain in value in the future, as the databases, annotations and statistical, bioinformatic, data mining and artifical intelligence methods for learning based on prior information continue to improve.

## Methods

### Reference data

As a reference data we have used 1667 healthy *in vivo *samples from GeneSapiens database [7] representing 44 different tissue types (Additional file 1) with 6290-17220 genes per sample. Varying gene number is depending on Affymetrix array generation used to measure the sample.

### Transforming the expression profile of query sample into compatible form

Gene expression data from the query sample to be analyzed against the reference data is transformed into compatible form by following procedure. MAS5 preprocessing algorithm and subsequent EQ transformation is applied as specified in Kilpinen et al. [7]. AGC correction method [7,21] is then applied for the sample. Gene and array generation specific correction factors needed in the AGC correction are fetched from the reference database [7].

### Calculation of gene expression density estimates

The density of expression values for each gene in each tissue type was calculated (Additional file 2A-B) as follows: For computational efficiency we used fast Fourier transformation based approximation to calculate kernel density estimates (R 2.7.2 [47]). Kernel densities were calculated by using Gaussian window with bandwidth selection given by Scott et al. [48] (R function bw.nrd). Density is estimated from 0 to maximum expression value in the entire dataset plus two times the highest bandwidth for that gene, with 512 equally spaced points.

The modality of gene expression estimates was calculated by searching for peaks having at least 0.1 of the total area of the density estimate. 14% of the genes were excluded from the analysis primarily due to the ambiguous modality of expression distributions.

### Comparing a single query profile to the reference data

Gene and tissue specific expression density estimates (Additional file 1) are used to calculate the likelihood of obtaining the expression values observed in the query profile from each tissue type for gene *g *in tissue *t *as follows:

The value of the density diagram for gene *g *in tissue *t *corresponding to the expression value of gene *g *in the query sample is determined. Then that density value is compared to the density values of the 512 evaluation points of the density diagram of gene *g *in tissue *t *and the fraction of lower density values is calculated. This is called the tissue match score (tm-score), with 1 meaning perfect match between the query and tissue for expression of gene *g *and 0 meaning expression of the gene in the query profile is outside the observed expression range of gene *g *in tissue *t*. This calculation is repeated for each gene of the query profile against the density estimates of the same genes in each tissue type of the reference data. The calculations are detailed in Equation 1. Based on the tm-scores the expression values of genes of query samples are also classified typical or atypical for each of the reference tissues. This is done by determining the tm-scores for all evaluation points, and weighting the abundance of that tm-score by the value of the density diagram at that point. This is repeated for all genes in all tissues. It essentially leads significance value of the tm-scores (less than 5% likelyhood of having at least equal tm-score by chance when comparing samples of the tissue against itself).

For the purpose of defining the similarity of the query sample at the level of tissues we calculate a tissue specificity score (ts-score) for each gene in each tissue (Equation 2). The ts-score for gene *g *for tissue *t *is the mean of the ratio weighted differences of tms(*g*, *t*) and all tms(*g*, not *t*). This gives us a score that indicates how well the tm-score of *g *categorizes the query sample into *t*. The ratio weighing is done so that the larger the ratio of the tm-scores, the higher the resulting ts-score will be. For example, a tm-score of 0.6 is deemed to better differentiate from a tm-score of 0.2 than a score of 1 from 0.6, even though their differences are the same. The scaling is controlled by the scaling factor (□), which was set to 0.25 for the analyses in this paper. It produces scores of 1/2 to 5/6 with a difference of 0.5. Setting □ closer to 0 gives more weight to the ratio, whereas a larger value decreases it. See Equation 2 for details. Ts-score varies between 1 and -1 and describes how well gene *g *classifies the query profile into tissue t. A score of 1 means the gene has a unique level of expression in the tissue and the query profile has expression level matching it perfectly. 0 means that the expression level observed in the query sample cannot differentiate the tissue from other tissues. -1 means gene has a unique level of expression for the tissue and the query profile does not have that specific expression level.

The mean of tissue specificity scores (Equation 3) is used as similarity score at the tissue level.

Equation 1

Equation 2

Equation 3

An R implementation of the AGEP algorithm is available at https://github.com/skilpinen/AGEP

### Leave-one-out cross-validation (LOOCV)

In order to validate the accuracy of the method we performed leave-one-out cross-validation using 1667 healthy samples from the reference data. Density estimates for the tissue from which the query sample was removed were recalculated, and then the query sample was aligned to the tissues. From the results we calculated accuracy of identifying correct tissue type as first hit (Figure 1) and distribution of first and secondary hits per each tissue (Additional file 5). The sensitivity and specificity for each tissue were calculated (Additional file 4) as follows: for tissue *t *true negatives (*tn*) were non-*t *tissue samples that matched non-*t *tissues, false negatives (*fn*) were tissue *t *samples that matched a non-*t *tissue, true positives (*tp*) were tissue *t *samples that matched *t *and false positives (*fp*) were non-*t *tissue samples that matched *t*. Sensitivity was defined as *tp*/(*tp *+ *fn*) and specificity as *tn/*(*tn *+ *fp*).

In nearest-neighbor classification method the average expression of each gene in each tissue was calculated to form tissue average profiles. Samples were classified as the tissue having smallest Euclidean distance to the sample in question. A separate classification was made by classifying samples to the tissue with the highest Pearson correlation coefficient. In all cases, the sample in question was excluded from the calculation of average profiles.

With SVM we used libsvm package through R library e1071, with radial kernel. Since SVM cannot effectively handle missing values we imputed missing values to the data by using median value of data points in the tissues for the gene in question. Imputation was done for each tissue separately so that each missing value was replaced by median non-missing values. If all samples of a tissue had missing value then the gene was discarded from the analysis. This resulted in 11834 genes with no missing values for each of the 1667 samples. Imputing missing values for SVM lowers variation within the tissue and thus to some degree artificially enhances the performance of SVM, which was tested with 10-fold cross validation of the entire database.

### Independent validation with external dataset

External healthy *in vivo *samples used in additional independent validation were randomly selected from Array Express [1] study E-GEOD-7307. 250 healthy *in vivo *samples were selected, and of these, 195 samples were from tissues that were also present in the reference data, and were thus used for the validation.

All 195 samples were aligned against the reference data using AGEP, NN and SVM methods, as detailed above.

### Datasets used in testing individual samples

Hematopoietic stem cell sample and leukemic stem cell sample were acquired from Array Express [1] study E-GEOD-17054 (GSM426413.CEL and GSM426407.CEL, respectively) [49], AML and bone marrow granulocyte samples were from GEO [3] study GSE1159 [50] (GSM20692.CEL and GSM20971.CEL, respectively), Blood monocyte sample was from GEO study GSE1133 [18] (3AMH02082315_PB_CD14Monocytes.CEL). Both the granulocyte and monocyte samples were originally part of the reference database [7] but were excluded from the density calculations to be used as external samples. Myeloblast and monoblast samples were from Array Express [1] study E-GEOD-12803 [51] (E-GEOD-12803-raw-cel-1712284859.cel and E-GEOD-12803-raw-cel-1712284746.cel, respectively).

Duchenne muscular dystrophy samples were from Array Express [1] study E-GEOD-3307 [34].

Mesenchymal stem cell differentiation series was from Array Express [1] study E-MEXP-858. Within the study human mesenchymal stem cells, derived from bone marrow aspirations of iliac crest of healthy transplantation donors, were induced to differentiate into adipocytes with specific induction cocktail (described in detail in experiment description file E-MEXP-858.idf.txt available through Array Express).

### Gene set enrichment analysis

In order to define the similarity of the query sample and the tissues at the level of biological functions tissue match scores were analyzed in terms of a priori known gene sets. For each gene set the relative enrichment of the members of gene set among the atypical, for the tissue in question, part of the transcriptome was calculated. Gene sets were derived from molecular signatures database [52,53] and Panther database [54].

### Boxplots

In boxplots there is one box for each tissue of reference data. Lines signify median expression; boxes extend to 25 and 75 percentiles while whiskers extend to the 1.5*IQR. Data points beyond are shown as individual points. Number of data points for each tissue is shown in the parenthesis. Expression level of the gene in individual samples is shown only as line after data of the reference database.

### Tissue tree

The phylogenic tree for the tissues in the reference database was calculated as follows: the density estimates for a gene in one tissue was compared to the density estimate for the same gene in another tissue. The area of the non-overlapping part was calculated. This was done for all genes that had density estimates in both tissues. The distance between two tissues was set as the median of the non-overlapping areas of all their common genes. The tree was calculated using the hclust() R function with the linkage parameter of "complete".

## Abbreviations

TMS: Tm-score: Tissue match score; TSS: TS-score: Tissue specificity score; AGC: Array-generation based Gene Centering; NN: Nearest neighbor; SVM: Support vectore machine; MSC: Mesenchymal stem cell; LSC: Leukemic stem cell; HSC: Hematopoietic stem cell; AML: Acute myeloid leukemia.

## Authors' contributions

SK contributed to the concept of alignment of expression profiles, invented and implemented primary methodology and primarily wrote the manuscript. KO contributed to the mathematics of the methodology, performed several validations and participated in the manuscript writing. OK supervised the entire project and participated in manuscript writing and editing. All authors read and approved the final manuscript.

## Conflict of interest statement

S.K. and K.O. are inventors on a patent application regarding this method. S.K. and O.K: are shareholders in Medisapiens Ltd., which develops microarray data analysis technologies.

## Supplementary Material

Additional file 1 **A number of samples in the reference data for each tissue class**. A number of samples in the reference data for each tissue class. Click here for file

Additional file 2 **Schematic diagram illustrating the estimation of gene expression densities**. A) Measured expression levels of a gene in five tissues, 50 samples per each tissue. B) Density values for expression of the gene in five tissues across entire observed expression range (from 0 to maximum) as estimated with 512 equally spaced points. The area of each density estimate is normalized to 1. C) A boxplot representation of expression levels of a gene across various tissues and in 2 individual test samples (the two rightmost entries). With the AGEP method Sample A (highlighted in red) gets high tm-scores (close to 1) for the gene in question for the majority of tissues having a similar very low expression level (such as mesenchymal stem cell), somewhat lower tm-scores for example against skin and bone and very low (close to zero) for tissues like adipose tissue. Sample A also gets ts-scores close to zero for the majority of tissues (there are no tissue specific expression levels for a majority of tissues) but close to -1 for adipose tissue. This -1 is because the expression in adipose tissue for the gene in question is nearly unique (adipose tissue is very nearly the only tissue only with expression levels above 3800). Sample B (highlighted in blue) gets a low tm-score for majority of tissues as the expression level of this gene in the sample does not match the low expression levels observed in the majority of tissues. However, sample B gets a very high tm-score (close to 1) for adipose tissue as it perfectly matches the expression levels observed in that tissue. Also, as the expression levels for adipose tissue are tissue specific, sample B gets a very high (near 1) ts-score for adipose tissue. Click here for file

Additional file 3 **Results of the classification accuracy of the AGEP algorithm across all tissue types**. A) Fraction of the samples from each healthy tissue type, where AGEP correctly defined the *a priori *known tissue type in leave-one-out cross validation B) Fraction of samples from each healthy tissue type using external samples where AGEP correctly classified the exact tissue of origin, where the classification resulted in a biologically relevant tissues (e.g. match to same organ but on different level of annotation), or a wrong match C) Summary of accuracy of finding *a priori *known tissue type as primary match over all 195 tested samples Click here for file

Additional file 4 **Specificities and sensitivites of AGEP**. Specificities and sensitivities of the AGEP method in identifying each tissue in LOO analysis. Click here for file

Additional file 5 **Results of leave-one-out validation**. Results of leave-one-out validation of tissue match accuracy of entire reference data. Distribution of primary and secondarily matching tissue types as fractions of samples of each tissue type. Click here for file

Additional file 6 **Results of tissue match accuracy of external samples**. For each randomly chosen sample the primary match is shown as well as classification whether it was perfect match, similar match or incorrect match. 29 samples were censored from the analysis with due to missing reference tissue or due to ambiguous original annotation. Click here for file

Additional file 7 **Alignment of Duchenne samples**. A) Alignment results of ten duchenne patient samples at the level of tissues (five best matching tissues are shown) B) Expression profile of ADIPOQ, a known adipose tissue specific gene, across the reference data and ten duchenne patient samples. Click here for file

Additional file 8 **Differentiation time series results**. Results of applying array alignment tool for differentiation serie of mesenchymal stem cell at the tissue similarity level. Between timepoints of 0 h and 3 h all replicates (A, B and C) show highest similarity with mesenchymal stem cells and only slight increase in similarity with adipose tissue. At 9 h time point similarity with mesenchymal stem cells begins to decrease. At 7d timepoint cells no longer have transcriptomic profile of mesenchymal stem cells and have more increased similarity with adipose tissue and heart. Click here for file
